# Lymphatic Tissue Transfer: Ultrasound-Guided Description and Preoperative Planning of Vascularised Lymph Nodes, Lymphatic Units, and Lymphatic Vessels Transfers †

**DOI:** 10.3390/jpm12081346

**Published:** 2022-08-21

**Authors:** Giuseppe Visconti, Alessandro Bianchi, Marzia Salgarello, Alba Di Leone, Akitatsu Hayashi, Riccardo Masetti, Gianluca Franceschini

**Affiliations:** 1 Dipartimento per la Salute della Donna, del Bambino e di Sanità Pubblica, Fondazione Policlinico Universitario “Agostino Gemelli” IRCCS, Università Cattolica del “Sacro Cuore”, Largo A. Gemelli 8, 00168 Roma, Italy; 2 Dipartimento di Scienze della Salute della Donna, del Bambino e di Sanità Pubblica, Fondazione Policlinico Universitario “Agostino Gemelli” IRCCS, Università Cattolica del Sacro Cuore, 00168 Roma, Italy; 3 Center for Lymphatic Surgery, Kameda Medical Center, Kamogawa 278-8510, Japan

**Keywords:** lymphedema, lymphatic surgery, super microsurgery, ultrasound, microsurgery, perforator flap

## Abstract

Background: The modern concept of lymphatic transfer includes vascularised lymph node transfer (VLNT), lymphatic vessels transfer (lymph interpositional flap transfer, LIFT) and lymphatic system transfer (vascularised lymph nodes and afferent lymphatics, LYST). The aim of this paper was to report our experience with different types of lymphatic transfer. Patients and Method: From June 2016 to June 2020, 30 consecutive patients affected by extremity lymphedema and 15 patients affected by post-traumatic lower extremity soft tissue defects, underwent lymphatic transfer (VLNT, LYST or LIFT). All cases were preoperatively evaluated by both high frequency and ultra-high frequency ultrasound. Flap features were recorded, as well as qualitative and quantitative outcomes at 1 year postoperative. Results: The mean postoperative lymphedema index reduction was 7.2 ± 5.7 for upper extremity and 20.7 ± 7.1 for lower extremity. No dismission of compression garments occurred, 12 patients (26%) referred more stable results of physical treatment and 1 case showed a 1-class compression reduction. All the LIFTs aimed as preventive did not develop post-traumatic lymphedema. In all cases of distal placement of VLNT and/or LYST, patients were dissatisfied with the aesthetic appearance of reconstruction, while no donor site scar disappointment was referred. Conclusion: When LVA is not feasible, LTT may represent a valid treatment option. This report was aimed at comprehensively describing and assessing how different lymphatic tissue transfer modalities may fulfil the different reconstructive needs of lymphedema patients.

## 1. Introduction

The idea of transferring lymphatic tissue to bridge a lymphatic gap and restore the drainage function of lymphedematous limbs dates back to the 1920s [1], and its first clinical application, more than half a century later, featured as a vascularised lymph node transfer (VLNT) [2]. This is a physiological procedure in which functional lymph nodes, along with their vascular pedicle, are harvested as a flap and transferred to a lymphedematous recipient site.

The introduction of VLNT largely anticipated the modern knowledge of anatomy, physiology and pathology of the lymphatic system, and the advances in its comprehension over recent decades have led to a progressive evolution of the concept of lymphatic tissue transfer.

The mechanism of action of VLNT is incompletely understood, being most probably the combination of two hypotheses: bridging and pumping functions [3]. The first relies on the regenerative abilities of the lymphatic system through lymphangiogenesis, whereby new lymphatic collateral pathways form under the stimulus of the transplant via VEGFR-C [4], and connect the VLNT to the surrounding severed lymphatic vessels and nodes of the recipient site [5]. The lymph, thus, leaves the VLNT through the efferent lymphatics, and the nodes represent a bridge between the proximal and distal lymphatics of the recipient site. The pumping hypothesis is based on the evidence that lymphovenous communications exist within the transplant [5], or they are established via neo-lympahngiogenesis [3], and act as a way out for the lymph. The nodes, therefore, work as sponge or even as lymph pumps, as they are endowed with spontaneous contractility, absorbing the fluid from the interstitium of the recipient site and ejecting it into the venous circulation [6,7,8].

Along with the discoveries on VLNT function, it clearly emerged that a key to success was that, compared with previous non-vascularised node transfers, it preserves the lymphatic vessels not merely as conduits but also as functional vessels that represent the drainage route of the lymph out of the lymphedematous limb [5].

After a better understanding of lymphatic response to injury and regeneration [9], it has been demonstrated that the lymphatic flow can be restored after tissue replantation or free flap transfer without supermicrosurgical lymphatic vessels anastomosis or lymph node transfer, by respecting the compatibility of lymph axiality [10]. The lymphatic collectors assumed a role as a transfer of lymphatic tissue, and the transferring of lymph node together with their afferent lymphatics to enhance the mechanisms of action of VLNT has been applied in clinical practice [11].

**Figure 1:**
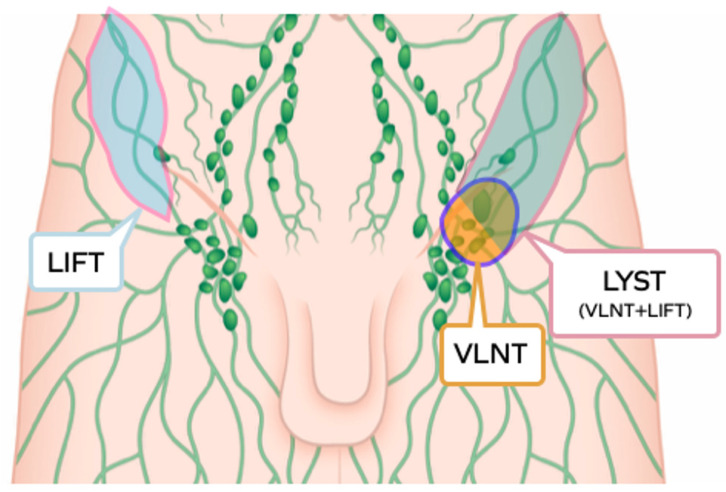
Graphic illustration of the three subtypes of lymphatic tissue transfer: LIFT, lymphatic interposition flap transfer; VLNT, vascularised lymph node transfer; LYST, lymphatic system transfer.

All the components of the lymphatic system, lymph capillaries, lymph collecting vessels and lymph nodes, have been shown to represent an active tissue able to interact with the recipient site, and to be part of the modern, all-encompassing concept of lymphatic transfer, including vascularised lymph node transfer (VLNT), lymphatic vessels transfer (lymph interpositional flap transfer, LIFT [11]) and lymphatic system transfer (vascularised lymph nodes and afferent lymphatics, LYST [12]) (Figure 1).

The aim of this paper is to report our experience with the different types of lymphatic transfer, for which no comprehensive description is available in the literature, and to describe the use of ultrasound (US) in the preoperative planning of these composite flaps.

## 2. Patients and Methods

From June 2016 to June 2020, 30 consecutive patients affected by extremity lymphedema, and 15 patients affected by post-traumatic lower extremity soft tissue defects, underwent lymphatic transfer (VLNT, LYST or LIFT) at Fondazione Policlinico Unversitario “Agostino Gemelli” IRCSS, in Rome. All lymphedema patients referred refractoriness to physical treatments and were on compression bandage treatment. The indication to lymphatic transfer in lymphedema patients was the presence of significant segmental dermal backflow together with the impossibility of identifying functional lymphatic pathways on lymphoscintigraphy, indocyanine green lymphography (ICG-L), and ultra-high frequency ultrasound (UHF-US). All cases were preoperatively evaluated by two types of US: US using an 18-MHz linear probe (MyLab 50 X-Vision; Esaote, Genoa, Italy), and UHF-US using 48-MHz and 70-MHz linear probes (Vevo MD; FUJIFILM Visualsonics, Toronto, ON, Canada) in B-mode and colour Doppler mode. 

### 2.1. Vascularised Lymph Node Transfer

The VLNT included the inguinal, supraclavicular and lateral thoracic donor sites. 

**Figure 2:**
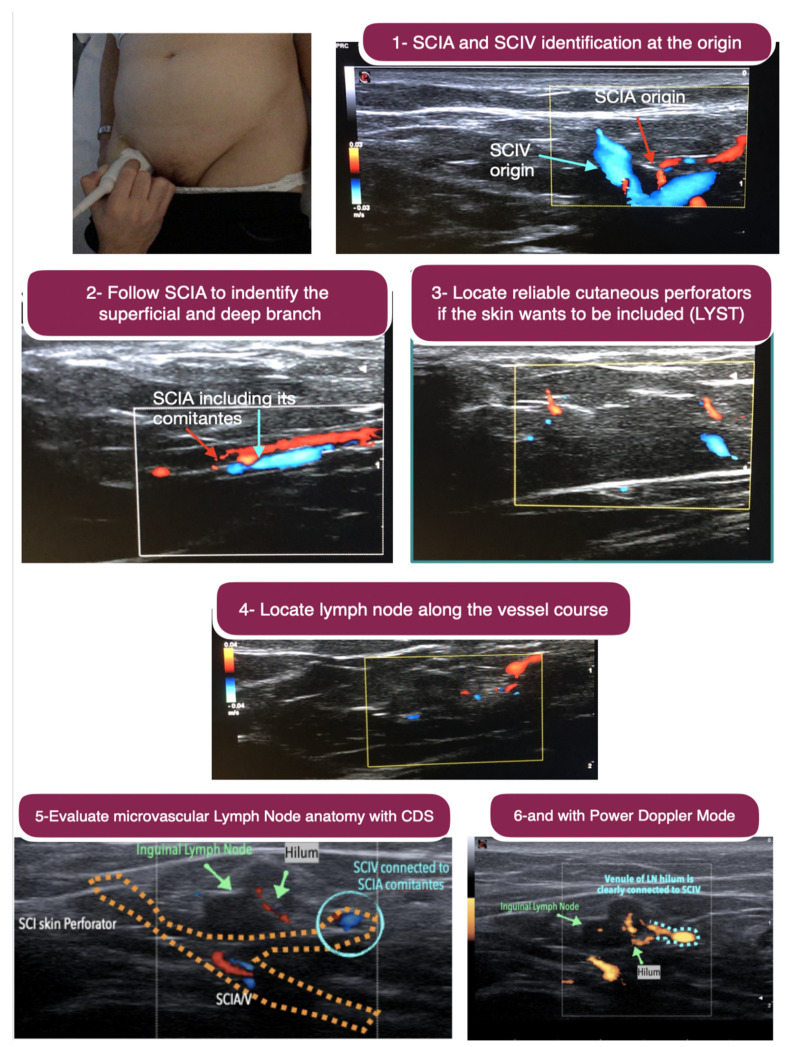
Step-by-step ultrasound preoperative planning of inguinal vascularised lymph node transfer. **Above, left**: The 18-MHz linear probe is used. **Above, right**: Step 1. Identification of the superficial circumflex iliac artery and its comitantes veins (SCIA) as well as superficial circumflex iliac vein (SCIV) at their origin (i.e., femoral artery and vein). **Second row, left**: Step 2. The SCIA is followed to identify the superficial and deep branch. **Second row, right**: Step 3. Location of reliable cutaneous perforator from the selected branch (superficial or deep) if the skin wants to be included in the flap. **Third row**: Step 4. Locate lymph nodes along the vessel course. The superficial branch is usually the branch with most nearby lymph node. **Below, left**: Step 5. Once the lymph node is identified, the microvascular anatomy is evaluated using the colour-duplex mode to understand the lymph node arterial input and venule output. In some cases, the venule output belongs to SCIV rather than SCIA comitantes, and this information is very valuable in flap planning and execution. **Below**, **right**: When the colour-duplex mode is not very clear, the power doppler mode can be employed for clearer evaluation. Comparing the last two images, it is clear that the power doppler is more powerful in seeing that the venule output of the lymph node is clearly connected to SCIV.

The superficial inguinal nodes were harvested combined with free abdominal tissue transfer (DIEP), both as one single flap and as two independent flaps. Using the 18-MHz, the superficial circumflex femoral and the superficial inferior epigastric vessels were located and evaluated for calibre and for the presence of lymph nodes along their course. The selected pedicle was further studied using the 48- and 70-MHz probes to evaluate the depth of the superficial and Scarpa fascia, the thickness of the adipose tissue layers, and the vascular arborisation throughout the adipose tissue to the superficial lymph nodes, the latter not always clearly discernible with an 18-MHz probe. The nodes were evaluated in terms of number and dimensions, and the location and course of the main afferent and efferent collectors were identified with the 48- and 70-MHz probes, so to harvest the nodes maintaining their lymphatic continuity and vascularity (Figure 2).

In all cases, preoperative lymphoscintigraphy and intraoperative gamma probe were used to detect and spare the sentinel nodes of the lower extremities. The flap harvest was performed after intradermal injections of Patent Blu to allow easier identification of lymph nodes and lymphatic collectors, without interfering with the evaluation of flap viability by fluorescence angiography. When the LNF was harvested independently of the DIEP flap, perforators to the overlying skin were included to allow the transfer of a monitor skin paddle. The recipient site was the axilla in all cases, associated with an extensive scar release to create space, decompress the vascular structures and identify the recipient vessels.

The right supraclavicular lymph nodes were harvested in a compartmental fashion, according to the anatomy of the supraclavicular region that shows two distinct compartments: the superficial lymph nodes along the external jugular vein, responsible for the skin drainage of the posterior scalp, and the deep lymphatic tissue that drains both the head and neck skin, as well as the oral cavity, thyroid gland, lungs and breast [13]. Therefore, two independent lymph node flaps (LNF) were raised from each donor site: a venous flow-through LNF based on the external jugular vein, which included the skin lymphatic system in the superficial compartment, and an LNF based on the traverse cervical artery and vein containing the deep lymphatic compartment. In all cases, the day before surgery, right upper limb reverse mapping was performed by ICG-L to exclude the supraclavicular nodes to drain the upper limb. The left donor site was not considered because of the potential risk of thoracic duct injury. An 18-MHz probe was used to evaluate the transverse cervical vessels and the jugular vein, the fascial plane dividing the superficial and deep lymphatic compartments (supraclavicular nerves laterally and the middle cervical fascia medially), while 48- and 70-MHz probes were employed to further investigate the microvascular and lymphatic anatomy as described for the inguinal nodes. All supraclavicular flaps were employed for lower extremities lymphedema. The superficial compartment LNF was anastomosed in a flow-through fashion along the course of the great saphenous vein in the knee region, while the deep compartment LNF was anastomosed in an end-to-end fashion to the medial sural artery and comitantes veins.

**Figure 3:**
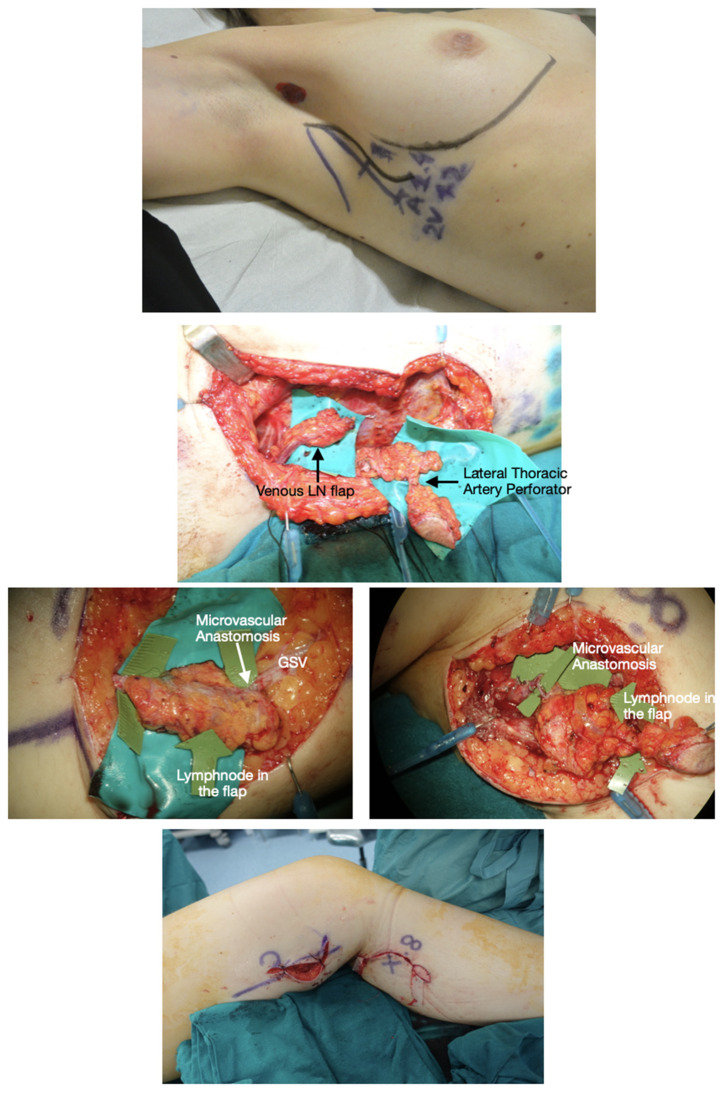
Lateral thoracic VLNT (LT-VLNT). **Above**: Preoperative planning of LT-VLNT using the 18-MHz linear probe and ICG-lymphography of the lateral thoracic region. Notice the black dot in the axilla showing the node marked by reverse mapping using lymphoscintigraphy. The incision is planned not to alter the breast aesthetic as a vertical lazy-S. The inframammary fold is marked to avoid any violation of this important aesthetic structure. **Second row**: Dual LT-VLNT has been harvested. The uppermost flap is a venous lymph node flap based on the thoracoepigastric vein. The lower VLNT flap, including a monitor skin paddle based on a perforator marked preoperatively, is based on the lateral thoracic artery, comintantes vein and thoracoepigastric vein lower portion. **Third row, left**: The venous VLNT is anastomosed in a flow-through fashion to the great saphenous vein above the knee. The green arrows show lymph nodes in the flap. **Third row, right**: The arterovenous VLNT is anastomosed to medial sural vessels in the calf area. **Below**: Final inset at the end of surgery. The venous flap is buried and monitored in the postoperative time with ultrasound, and the arterovenous flap has a small skin-paddle used for monitoring.

The lateral thoracic LNF was harvested based on the lateral thoracic vessels as for the traditional technique, after reverse lymphatic mapping to avoid the lymphatic drainage of the upper extremity (Figure 3). The same preoperative evaluation using US and UHF-US were performed as for the other VLNTs. 

### 2.2. Vascularised Lymph Nodes and Afferent Lymphatic Vessels Transfer

**Figure 4:**
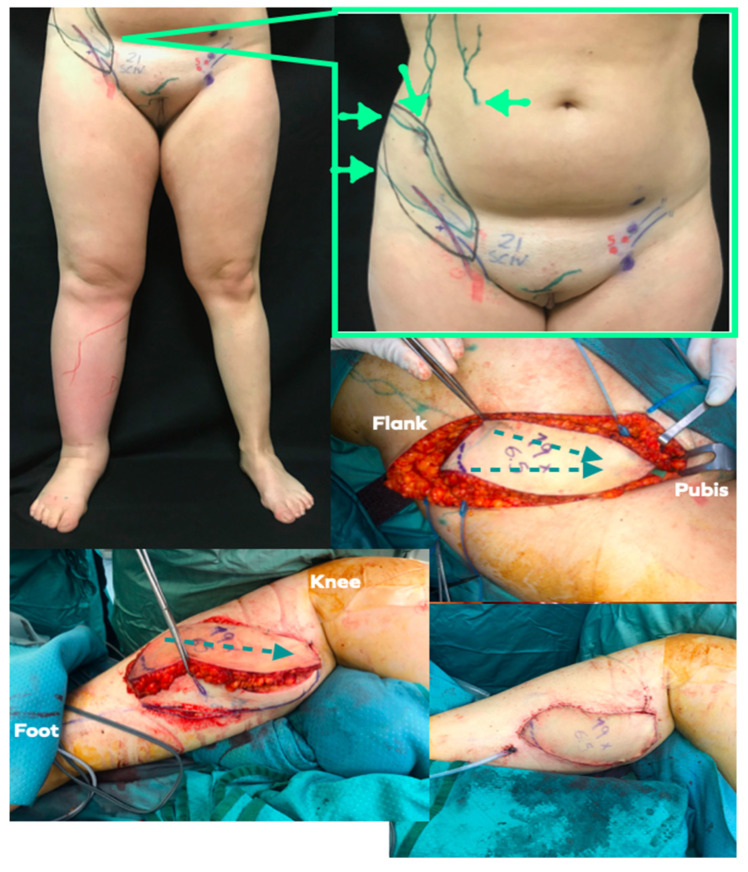
LYST case example. **Above, left**: Preoperative picture of 30-year old female patient suffering from primary lower limb lymphedema. Lymphoscintigraphy showed dermal backflow in the no tracer progression. **Above, right**: Preoperative planning markings were conducted with US and ICG-lymphography. The flap (flap margin is marked in black) has been oriented along the main axis of lymphatic channels (green lines) and SCIA (blue and red lines). Green arrows show the ICG injection points to highlight the lymphatic channels draining toward working inguinal nodes (green dots). **Middle, right**: Flap elevated according to preoperative planning. The green dotted line shows the lymph axiality as already highlighted preoperatively. **Below, left**: The flap has been anastomosed to the medial sural vessel and inset respecting lymph axiality. Lymphedematous skin and soft tissue have been resected to introduce more healthy soft tissue (i.e., flap) and resect the lymphedematous tissue. **Below, right**: Final picture.

The transfer of vascularised afferent lymphatics together with their lymph nodes, which are also vascularised, have recently been popularised as lymphatic system transfer (LYST) [11]. These flaps were all harvested from the groin region. The preoperative ICG-L was employed to map the lymph collecting vessels draining the lower hemiabdomen and the flank up to the inguinal nodes. The HF-US, using the 18-MHz probe, was used to evaluate the superficial circumflex iliac artery (SCIA) and superficial vein (SCIV), as well as the superficial and deep branches of the SCIA, and to select the best vascular pedicle. The UHF-US allowed clarification of the characteristics of the lymph nodes and their afferent lymphatics, to assess the calibre and functionality of the collectors identified by ICG-L, as well as to exactly locate the nodes to which they were connected, which was not easily discernible by ICG. The flap was then designed according to the axiality of both the vascular pedicle and the afferent lymphatics, so to include the highest number of afferent collectors to the selected nodes. The precautions to avoid iatrogenic lymphedema were the same as for the inguinal VLNT. LYST flaps were used as free flaps for upper extremity lymphedema, both in the proximal and distal region, while for the lower extremity, were placed at the calf level (Figure 4). 

### 2.3. Vascularised Lymphatic Vessels Transfer

**Figure 5:**
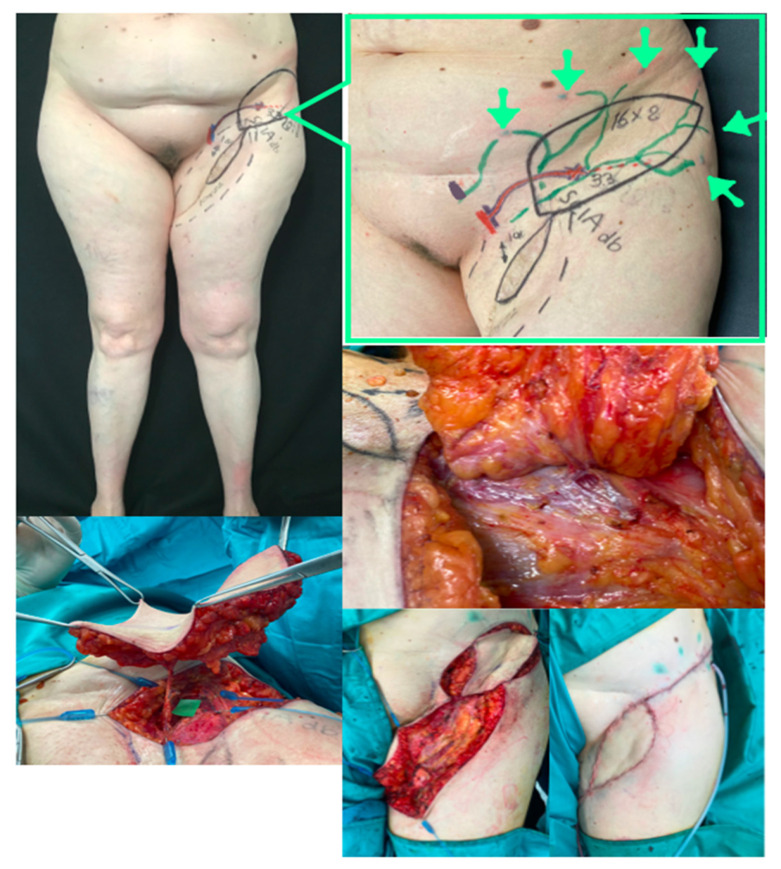
LIFT case example. **Above, left**: Preoperative picture of 57-year old female patient suffering from a retracted inguinal and upper thigh scar three years after inguinal dissection for melanoma of the foot. Lymphoscintigraphy showed dermal backflow in the upper inner thigh. **Above, right**: Preoperative planning markings performed with US and ICG-lymphography. Notice that in addition to the long inguinal scar, we found that the SCIA deep branch was spared, travelling below the muscular fascia on top of sartorius muscle and perforating the fascia at the marked point. Perforator calibre was 3.3 mm (probably due to delay phenomenon connected to ligation of other superficial vessels during inguinal dissection). Green arrows show the ICG injection points to highlight the lymphatic channels draining in some residual upper deep pelvic–inguinal lymph nodes. The skin paddle is marked in black. **Middle, right**: Flap elevation shows that the big perforator matches exactly with preoperative planning in terms of position, fascia perforation point and calibre. **Below, left**: The flap is elevated and the SCIA dissected up to its origin. **Below, right**: The thigh scar was revised and the flap rotated and transposed for final inset.

**Figure 6:**
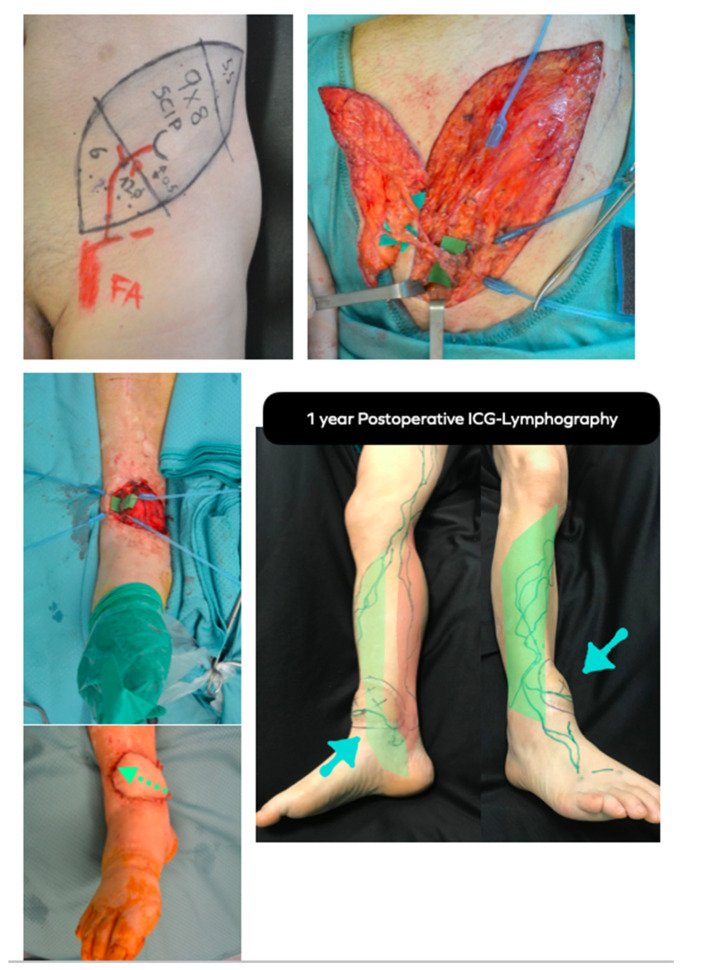
LIFT case example. **Above, left**: Preoperative planning of super-thin SCIP flap conducted with HF-US and UHF-US. **Above, right**: The super-thin SCIP flap has been elevated on superficial SCIA branch and SCIV. **Middle, left**: Intraoperative picture of the trauma-related ankle defect to reconstruct. Anterior tibial vessels have been exposed. **Below, left**: The SCIP flap has been inset according to lymph axiality (green dotted arrow). **Below, right**: 1 year postoperative ICG-lymphography showing that the flap functioned as a bridge between the anterolateral (green) and anteromedial (red) lymphatic pathways. Notice the lymphatic channel within the flap, marked in green. Blue arrows point to the flap.

The transfer of skin flaps aimed at transferring the lymph collecting vessels inside them, recently named as lymph interposition flap transfers (LIFT) [12], as well as vascularised lymph vessel transfer (VLVT) [14], were harvested as SCIP flaps in all cases. These were used as pedicled flaps for the treatment of lower extremity lymphedema associated with the removal of inguinal retracting scars after inguinal lymph node dissection, as free flaps for the reconstruction of soft tissue defects in lymphatic-rich areas with the aim of also preventing the occurrence of post-traumatic lymphedema. As free tissue transfers, the SCIP flaps were harvested as full-thickness, thin and super-thin flaps, depending on the resurfacing needs of the recipient site and the features of the lymphatic tissue. The preoperative ICG-L was used to map the lymphatic vessels and assess the lymph-axiality of the SCIP area, as for the other inguinal lymphatic transfers. The HF-US, using the 18-MHz probe, was used to evaluate the superficial circumflex iliac vessels and branches, evaluate the calibres and the course in the adipose compartments of the inguinal region. The selected pedicle, either superficial or deep SCIA branches, was further investigated with the 48- and 70-MHz probes to evaluate the vascular arborisation according to the UHF-US perforator classification [15]. The type 1 perforators, that take a direct course from the emergence to the dermis, preserving their calibre until the superficial fascia where they typically branch, were selected for super-thin and pure skin flaps [15], while the type 2 perforators, that preserve their calibre up to the Scarpa fascia, where they begin to arborise into collateral vessels and pass through the Camper fascia via smaller branches, were selected for thin flaps. The UHF-US allowed assessment of the presence of main lymphatic collectors within the superficial and deep adipose compartments, so for each case, the best flap according to the vascular and lymphatic characteristics was designed. The insetting in the donor site for pedicled and free flaps was performed by respecting the axiality of lymphatic vessels to increase the chance of lymph flow restoration. In the case of pure skin flaps, as the lymphatic transfer is represented by lymphatic capillaries, the lymph-axiality concept is not taken into consideration. LIFT flaps were used for lower extremities, for the treatment of lymphedema as pedicled flaps, and as free flaps for the prevention of lymphedema when reconstructing soft tissue defects (Figure 5 and Figure 6).

For all lymphatic transfers, patients were instructed to maintain the operated limb at rest for 20 days after surgery, and were referred to physiotherapy consultation at our centre 21 days after surgery. After that, patients followed their postoperative program with home-based lymphedema physiotherapists. Regular follow-up was performed in our outpatient clinic, and qualitative and quantitative outcomes were evaluated at 1 year follow-up.

## 3. Outcome Analysis

Patients’ demographics, intraoperative findings, and postoperative outcomes were recorded. The features of transferred lymphatic vessels and nodes were annotated for each case according to the lymphoscintigraphic, ICG-L, HF-US and UHF-US findings. Quantitative outcome analysis was performed by comparing pre- and postoperative upper extremity lymphedema (UEL) and lower extremity lymphedema (LEL) indexes [16,17]. Qualitative outcomes analysis was performed by reporting the pre- and postoperative need for compression garment and the compression class used according to RAL-GZG standard, and the patients’ subjective perception in skin stiffness and whole limb heaviness. One year after surgery, ICG-L and US evaluations were conducted.

Categorical variables are presented in numbers and percentages, continuous variables are presented by means and standard deviation. The Fisher’s exact test was performed for categorical variables, the independent samples *t*-test for continuous variables. Significance was defined as *p* < 0.05 (two sided).

## 4. Results

The 45 patients included 11 cases of upper limb lymphedema (UEL) (all secondary to breast cancer treatments) and 15 cases of lower limb lymphedema (LEL) (4 cases were primary and 11 were secondary to pelvic cancer or melanoma treatments). Fifteen patients underwent immediate soft tissue and lymphatic reconstruction for trauma. There were 33 women and 12 men, whose average age was 54.6 years (range 23–72 years) and the average body mass index was 27.34 kg/m^2^ (range 18.5 to 31.4). No patient was a smoker. 

All lymphedema patients had advanced stage disease, out of which 22 were rated as ISL 2b, and 8 patients were rated as ISL 3 (Table 1).

The VLNT were 27, including 11 inguinal, 13 supraclavicular and 3 lateral thoracic LNF. The inguinal VLNT were harvested together with DIEP flap for breast reconstruction in six cases, while two independent flaps (DIEP and Inguinal VLNT) were harvested in five cases. The recipient site was the axilla, the VLNT were anastomosed to recipient collaterals of the axillary or subscapular vessels; in the cases with the same vascular supply of the DIEP flap, this was anastomosed to the internal mammary vessels, and the SIEV was added as additional venous discharge for the LNF.

The LYST were seven, all from the groin region. For the LEL, the recipient site was the calf area, with anastomosis to the medial sural artery and comitantes vein, and oriented according to lymph axiality. For the UEL, the LYST were placed proximally in the axilla and upper arm in four cases, in one case it was placed distally at the level of the wrist, with vascular anastomosis to the radial artery and vein, and in three cases to the calf area.

The LIFT were 11 SCIP flaps, including 7 free flaps and 4 pedicled flaps. The free transfers were three thin and four super-thin SCIP flaps. The superficial branch of the SCIA was selected as vascular pedicle in all cases. They were used to reconstruct soft tissue defects and prevent the occurrence of post traumatic lymphedema in lower limbs, and in one case, for an upper limb. The four pedicled SCIP flaps were based on the deep SCIA branch, rotated in a propeller fashion and transposed to the inner upper thigh to treat LEL after the removal of the inguinal scar in melanoma patients. In all cases, the postoperative course was uneventful, and all flaps survived (Table 2).

Globally considered, an improvement in both quantitative and qualitative outcomes were observed at 1 year follow-up. The mean postoperative lymphedema UEL and LEL index reduction was 7.2 ± 5.7 for UEL, and 20.7 ± 7.1 for LEL. An improved lymph flow and tracer appearance time at postoperative lymphoscintigraphy were recorded in all cases. The VLNF with axillary scar release were associated with an improvement in patients’ perception of skin texture and limb heaviness discomfort in all cases, and the same was appreciated for the pedicled LYST after inguinal scar excision. No dismission of compression garments occurred, 12 patients (26%) referred more stable results of physical treatment (6 LYST, 3 supraclavicular and 3 axillary VLNT) and 1 case (distal LYST for UEL) showed a 1-class compression reduction. All the LIFT aimed as preventive did not develop post-traumatic lymphedema (Table 3).

In all cases of distal placement of VLNT and/or LYST, patients were dissatisfied with the aesthetic appearance of reconstruction, while no donor site scar disappointment was referred.

## 5. Discussion

The modern concept of lymphatic flaps includes vascularised lymph nodes transfer (VLNT), lymphatic system unit transfer (LYST) [11] and lymph interpositional flap transfer (LIFT) [12]. VLNT is currently seen as the first feature with which the all-encompassing concept of lymphatic tissue transfer (LTT) came to clinical practice. Its supermicrosurgical physiological counterpart, the lymphaticovenous anastomosis (LVA), is intuitively effective and has rapidly gained worldwide acceptance [18], while the mechanisms behind the transfer of lymphatic tissues are much more complex, and the clinical improvements are not consistently documented [19]. On the other hand, the LVAs are effective only if functional lymphatics can be detected and, even if the new lymphatic imaging modalities allow an expansion of the indications to advanced cases [18,20,21,22,23,24], the absence of functional lymphatics is still possible, and LTT may represent the only treatment option [19].

VLNT can be harvested from several reported donor sites, either cutaneous (inguinal, submental, supraclavicular, and lateral thoracic) or visceral (gastroepiploic and jejunal). The most popular source of lymph nodes is the inguinal area, mainly for the well-concealed scar and the feasibility of combining it with free abdominal tissue transfer for breast reconstruction. It allows the inclusion of abundant surrounding soft tissue, that is useful when a moderate-to-large skin paddle is needed [3]; and reverse lymphatic mapping and use of intraoperative gamma probes make the risk of LEL relatively low [25]. The other cutaneous sources of nodes are generally preferred when an increased risk of iatrogenic LEL is present, or when the inguinal vascular pedicles are damaged [3,19]. Visceral VLNT are relatively more recent, introduced with the advantages of an absent risk of iatrogenic lymphedema and inconspicuous scars when harvested through laparoscopy. However, no one donor site has proved to be superior to the others in terms of efficacy [26], and the two mechanisms of action support the effectiveness of VLNT both cutaneous and visceral, as well as in its orthotopic and eterotopic placement within an extremity. The main reported advantages of orthotopic VLNT include the convenience of combination with breast reconstruction, the axillary scar release, and the acceptability of the aesthetic results. Heterotopic placement is indicated when lymphedema mainly affects the distal area of the upper extremity and for lower limbs, because it exploits gravitational force [3]. In the latter setting, the compartmental harvesting of dual VLNT from the supraclavicular area is useful to obtain two independent flaps from the same donor site [13].

The transfer of lymphatic system units (LYST) is defined as the transfer of vascularised afferent lymphatic vessels along with their draining vascularised inguinal lymph nodes. It is based on the consideration that the effects of VLNT are postponed until afferent and efferent lymphatics develop. Therefore, LYST, by transferring long afferent lymphatics, requires a lesser degree of lymphangiogenesis [11], which is a variable that escapes the surgeons’ control. LYST can be positioned in both the upper and lower part of an extremity, according to the clinical manifestations, as for VLNT. In our report it was preferred for patient candidates to VLNT for LEL, or for UEL without the need of breast reconstruction. The LTT had the best improvement in symptom relief but more studies are needed to confirm the promising results of this technique.

The LIFT, lymph interpositional flap transfer, has been introduced after the evidence that lymph flow can be restored after tissue replantation or free flap transfer without supermicrosurgical lymphatic vessel anastomosis or lymph node transfer, mediated by the connection of lymphatic vessels that originally existed in an amputee/flap and a recipient site, without the development of new lymphatic pathways [10]. Every skin flap contains lymph collecting vessels, and can be used to prevent extremity lymphedema if the concept of lymph axiality is respected. Full-thickness, thin and ultra-thin flaps can be harvested for this purpose, and also pure skin flaps, by means of the lymph capillaries contained in them, are able to restore the lymphatic flow of the donor site [26]. More recently, the use of LIFT was extended from the prevention of post-traumatic lymphedema to the treatment of chronic lymphedema, with promising results shown when applied to reconstructing genital elephantiasis after radical excision, both as super-thin and pure skin flaps [26]. We report the application of pedicled SCIP flaps to reconstruct the crural–inguinal area after retracting scar removal in melanoma patients, and our findings confirm these promising results.

The preoperative planning based on HF- and UHF-US allowed us to make a step forward in the design of LTTs, as the UH-US using the 18-MHz probe allows a detailed analysis of the vascular pedicles and lymph nodes along their course, and the UHF-US, with 48- and 70-MHz probes, with resolutions as high as 30 µm, allows a precise anatomical evaluation of vascular arborisation and lymphatic structures [27]. In VLNT, the lymph nodes can be evaluated in terms of number and dimension, their vascular supply can be clarified, and the main afferent and efferent lymphatics can be identified, to maintain lymphatic continuity within the flap. In LYST, the afferent lymphatic identified by ICG-L can be evaluated for calibre and functionality, and the collectors invisible to ICG-L can be located and included in the flap. The characteristics of lymphatic collectors are also extremely useful for LIST, wherein adjunct US allows the choice of perforator that best fits the flap thickness required [15,20], and the compatibility of flap thickness with the course of main lymphatic collectors can be assessed.

A better understanding of which lymphatic flap construct is more effective in improving lymphedema requires a larger clinical series with longer follow-up.

## 6. Conclusions

All the components of the lymphatic system, lymph capillaries, lymph collecting vessels and lymph nodes have been shown to represent an active tissue able to interact with the recipient site, and to be part of the modern all-encompassing concept of lymphatic tissue transfer (LTT), including VLNT, LYST and LIFT. When LVA is not feasible, LTT may represent a valid treatment option. This report was aimed at comprehensively describing and assessing how different lymphatic tissue transfer modalities may fulfil the different reconstructive needs of lymphedema patients.

## Figures and Tables

**Table 1:** Patient and Disease Characteristics.

**Overall No. of Patients**	**45**
**Age, years**	
Mean ± SD	54.6 ± 12.1
Range	23–72
**Sex**	
Male, *n* (%)	12 (27%)
Female *n* (%)	33 (73%)
**BMI, kg/m^2^ body surface**	
Mean ± SD	27.3 ± 3.1
Range	18.5 –31.4
**Cancer-related lymphedema *n* (%)**	26 (58%)
**Upper extremity lymphedema *n* (%)**	11 (42%)
**Lower extremity lymphedema *n* (%)**	15 (58%)
Prostate cancer *n* (%)	4 (27%)
Endometrial cancer *n* (%)	8 (53%)
Melanoma *n* (%)	3 (20%)
**Radiation Therapy *n* (%)**	23 (77%)
**Primary lower extremities lymphedema *n* (%)**	4 (8%)
**Preoperative ISL stage**	
2b *n* (%)	22 (73%)
3 *n* (%)	8 (27%)
**Trauma—Lower extremity soft tissue defect**	15 (34%)
**Defect location**	
Proximal third of leg *n* (%)	5 (33%)
Middle third of leg *n* (%)	4 (27%)
Distal third of leg *n* (%)	5 (33%)
Foot	1 (7%)
**Comorbidities in trauma patients**	
Diabetes Mellitus *n* (%)	1 (7%)
Hypertension *n* (%)	5 (33%)
Active Smoking *n* (%)	0
Peripheral vascular disease *n* (%)	1 (7%)

**Table 2:** Flap Characteristics.

Characteristic	VLNT			LYST	LIFT	
	Inguinal	Supraclavicular	Lateral Thoracic	Inguinal	Free SCIP	Pedicled SCIP
Overall no. of flaps	6 with DIEP 5 apart	13	3	7	7	4
Flap size						
mean± SD, cm	8.5 × 5.8 ± 2.1 × 1.3		7.2 × 3.5 ± 1.8 × 0.9	18.1 × 6.8 ± 3.5 × 1.5	15 × 5.5 ± 2 × 1.8	16.2 × 6.3 ± 3.5 × 2.5
Flap thickness mean, range, mm						
Full thickness 25, 20–29	/	/	/	7	/	4
Thin flap 16.4 9–28	/	/	/	/	3	0
Superthin flap 5.5 4–7	/	/	/	/	4	0
Recipient site						
Orthotopic	11, axilla	0	3	4, axilla	/	4, inguinal scar
Eterotpic	0	13, leg	0	1, wrist 3, leg	/	0
Complications	0	0	0	0	0	0
UEL index reduction mean ± SD	8.1 ± 4.5	/	5.6 ± 3.5	18.3	/	/
LEL index reduction mean ± SD	/	13.4 ± 8.3	/	23.3 ± 8.5	/	16.8 ± 6.8
Limb softness	+++	++	++	+	+	+++

+ moderate soft; ++ soft; +++ very soft.

**Table 3:** Quantitative and qualitative outcomes analysis.

Outcome	Analysis		Significance
Upper Limb Lymphedema index postoperative reduction	Comparison of ULL index pre and postoperative at 1 year follow-up	Pre: 195.8 ± 45.9 Post: 188.3 ± 39.4 Δ: 7.2 ± 5.7	*p* = 0.17 paired *t*-test
Lower Limb Lymphedema index postoperative reduction	Comparison of LLL index pre and postoperative at 1 year follow-up	Pre: 345.4 ± 53.7 Post: 324.5 ± 58.6 Δ: 20.7 ± 7.1	*p* = 0.18 paired *t*-test
Improved stability of results after physical therapy	Association between flap type (VLNT vs LYST-LIFT) and improved stability after physical therapy	VLNT: 3/21 LYST-LIFT: 9/24	*p* = 0.10 Fisher’s exact test
Reduction of compression class	Association between flap type (VLNT vs LYST-LIFT) and reduction of compression class	VLNT: 0/21 LYST-LIFT: 1/24	*p* = 0.99 Fisher’s exact test
Improvement of subjective symptoms perception	Association between the scar release performed with the flap and improvement of symptoms	Release +: 15/21 Release −: 4/9	*p* = 0.22 Fisher’s exact test

## Data Availability

Not applicable.
